# Tobacco use and chemosensory impairments among current adult tobacco users in the US: Data from NHANES 2013–2014

**DOI:** 10.18332/tid/94202

**Published:** 2018-09-18

**Authors:** Liane M. Schneller, Scott McIntosh, Dongmei Li, Irfan Rahman, Deborah Ossip, Maciej Goniewicz, Richard J. O’Connor

**Affiliations:** 1 Roswell Park Cancer Institute, Buffalo, United States; 2 University of Rochester, Rochester, United States

**Keywords:** smell impairment, taste impairment, smoking, menthol cigarettes

## Abstract

**INTRODUCTION:**

Among US adults 40 years and older, about 23% report problems with their ability to smell, and 19% report problems with their ability to taste. Chemosenses are a first line of defence against environmental hazards (e.g. fires and leaking gas). A potential risk factor of chemosensory disorders includes nicotine product use, such as cigarette use. This study aims to assess the relationship of taste and smell alterations with type of recent nicotine product use (e.g. inhaled versus smokeless), recent cigarette use, and mentholation status based on data from NHANES 2013–2014.

**METHODS:**

A total of 3186 men and women, 40 years and older, from NHANES 2013–2014 were assessed for smell and taste impairment, according to their recent nicotine product use. Taste impairment was identified as inability to identify quinine as bitter in the whole-mouth taste test. Impairment of smell was defined as failing to identify six or more of eight specific odors. Logistic regression models were adjusted for age, gender, and race/ethnicity.

**RESULTS:**

Approximately 13% of participants failed the smell examination. No significant association between smell examination outcome and recent nicotine product use was found, though recent cigarette use showed a trend toward positive association (OR=1.66, 95% CI: 0.76–3.63) and mentholation status showed a trend toward negative association (OR=0.57, 95% CI: 0.22–1.49) on smell examination results. About 17% of participants failed the taste examination, and trends toward positive association were seen between taste examination outcomes and both recent nicotine product use (OR=1.28, 95% CI: 0.99–1.65) and recent cigarette use (OR=1.30, 95% CI: 0.50–3.40).

**CONCLUSIONS:**

Findings indicate that recent use of nicotine products has an inconsistent relationship to dysfunctions in taste and smell. However, limiting the use of inhaled nicotine products, such as from cigarette use, could prove beneficial to a person’s taste and smell ability.

## INTRODUCTION

Among adults who are 40 years and older in the US, about 23% report problems with their ability to smell and 19% report problems with their ability to taste^1^. Although anatomically distinct, taste and smell are closely associated^2^. Many people who complain of taste impairment usually are also suffering from an impairment of smell^2^. Diagnoses with an impairment in taste or smell, such as phantosmia (smelling phantom odors) or dysgeusia (distorted sense of taste), are known as chemosensory disorders^1,3^. Taste and smell, or chemosenses, not only allow people to experience food flavors and pleasurable aromas, but affect nutrition and alert them to environmental

hazards, such as spoiled food, poisonous fumes, fires, and leaking gas^1,3-5^. Chemosensory disorders have been associated with aging, sinus and respiratory infections, poor oral hygiene and dental problems, head trauma, and as an iatrogenic effect of radiation therapy for head and neck cancers^6,7^. Another potential risk factor of chemosensory disorders is nicotine product use, primarily smoking.

Smoking is known to cause sinus and respiratory infections^8-10^, and delay recovery from head trauma^11^, which could lead to chemosensory disorders. Furthermore, smoking has been shown to alter taste buds and the vascularization of the fungiform papillae, diminishing the ability to taste^12,13^. A limited number of studies have looked at this association with inconsistent findings^4^. Smoking has been found to be associated with smell and taste impairments in some studies^4,14^, while others found no association^15^, or a small protective effect^16,17^. The mixed nature of the literature may be related to past exposure versus continued exposure - quitting for an extended period of time may allow the senses to recover from the effects of prior smoking^17^. However, other smoking behavior factors, such as concurrent use of other nicotine products or use of mentholated cigarettes, may influence chemosenses. Smokeless nicotine-product use may result in dental problems that could affect a person’s sense of taste^7,18^. Flavors can play an important role in shaping consumer perceptions of various types of products by creating the sensation of burning, heat, or coolness^19^. Menthol, in particular, is known for its cooling sensation, and it has been shown to create a purely subjective increase in nasal airflow and patency when added to chewing gum^20^. Menthol in cigarettes reduces the harshness of cigarette smoke by acting on receptors expressed on the trigeminal nerves of the nose and mouth^21^. Menthol cigarettes comprise more than 1/3 of the current market^22^ but to our knowledge, menthol cigarette use has not been examined specifically in the context of chemosensory deficits.

The National Health and Nutrition Examination Survey (NHANES) is designed to assess the health and nutritional status of the US adult and youth populations. The NHANES provides prevalence data for selected diseases and risk factors, including smoking and chemosensory problems. Using NHANES 2013–2014 data, this study aims to assess the prevalence of taste and smell alterations according to participants’ type of recent nicotine product use, recent cigarette use, and menthol cigarette use. To our knowledge, this is the first study using nationally representative taste and smell examination data to assess chemosensory impairment and its association with nicotine product use.

## METHODS

### Survey design and study population

The NHANES interviews and physical examinations are composed by the National Center for Health Statistics (NCHS) under the Centers for Disease Control and Prevention (CDC). About 5000 participants are examined each year to represent all ages of the non-institutionalized US population^23^. Hispanics, Asian, Non-Hispanic Black, low income, and persons 80 years of age or older were oversampled to provide precise estimates^23^. Each participant is assigned a sample weight to allow for the calculation of unbiased, nationally representative estimates^23^. Data from the NHANES 2013–2014 Recent Tobacco Use questionnaire, as well as the Taste and Smell Examination, both collected at the Mobile Examination Center, were analyzed in this study.

### Tobacco use

#### Self-reported tobacco use in the last 5 days

Use of nicotine products, including cigarettes, cigars, e-cigarettes, smokeless tobacco, pipe, hookah, snus, and dissolvable tobacco, in the last 5 days, was collected from participants 12 years and older^24^. Categories were created to denote if the nicotine products used in the last 5 days were inhaled (cigarettes, pipes, cigars, little cigars or cigarillos, water pipes, hookahs, or e-cigarettes), smokeless (chewing tobacco, snuff, snus, or dissolvables), a combination of inhaled and smokeless products or no nicotine at all. Categories were created taking into account serum cotinine levels using a cutoff level of 3 ng/mL, i.e. participants who had serum cotinine levels that did not match their self-reported nicotine product use in the last 5 days (e.g. participant reported not using any form of nicotine product in the last 5 days but had a serum cotinine level equivalent to that of a nicotine product user) were excluded from the analyses (N=269). In addition, among those who reported using a nicotine product in the last 5 days, cigarette use in the last 5 days, specifically, was assessed.

#### Mentholation status of regular brand of cigarettes

Participants who reported the use of cigarettes in the last 5 days were assessed if they had smoked 100 cigarettes or more in their lifetime. If they had smoked 100 cigarettes or more in their lifetime, responses were linked to data from the Cigarette Use Questionnaire^25^ to determine the mentholation status of their regular brand of cigarettes. If cigarette users in the last 5 days did not smoke 100 cigarettes or more in their lifetime, the mentholation status of their regular brand of cigarettes was not ascertained due to skip patterns in the Cigarette Use Questionnaire^25^.

### Chemosensation measures

#### Taste and smell examination eligibility criteria

Participants who were 40 years and older, and not currently pregnant or breastfeeding, participated in the NHANES taste and smell examination^26^. Participants who had had a skin rash or allergy due to quinine were excluded from the quinine taste test^26^. Furthermore, participants were presented with three lights with varying intensity (dim, moderate, bright)^26^. If participants were unable to correctly rank the three light intensity levels they were excluded from the taste test (but not the smell test), as it was assumed that they did not understand the procedures that were also used in the taste test^26^.

#### Smell examination

The smell examination consisted of an 8-item ‘scratch and sniff’ odor identification test that included: chocolate, strawberry, smoke, leather, soap, grape, onion, and natural gas^26^. Participants lightly scratched the odor test strip, smelled the odor, and were asked to identify the odor from a list of four possible responses^26^. The distribution of the smell exam scores was skewed to the left; therefore responses were scored as pass/fail, with impairment of smell defined as failing to identify six or more of the odors^14,27^.

#### Taste examination

The taste examination consisted of a tongue-tip taste testing, as well as a whole-mouth taste testing. For the tongue-tip taste testing, participants were presented with 1mM quinine, which is bitter, and 1M sodium chloride (NaCl), which is salty^26^. Each was applied to the tip of the tongue in a standardized manner^26^. While keeping their tongue out, participants identified the flavor as salty, bitter, sour, some other taste, or no taste^26^. The whole-mouth taste testing consisted of gently swishing 10 mL of a solution in the mouth for 3 s and then spitting it out^26^ . There were two randomized presentation orders of solutions: 1) 0.32M NaCl, 1mM quinine, 1M NaCl, and 2) 1M NaCl, 1mM quinine, 0.32M NaCl^26^. Participants were then asked to identify the flavor as salty, bitter, sour, some other taste, or no taste^26^. For both the tongue-tip taste testing and the whole-mouth taste testing, participants rinsed their mouths out with water in between tastants^26^. Taste impairment was identified as inability to identify quinine as bitter in the whole-mouth taste test^28,29^.

### Data and statistical analysis

Statistical analyses were conducted using SAS 9.4 (SAS Institute, Inc., Cary, North Carolina) and SUDAAN 11.0.1 (RTI International, Research Triangle Park, North Carolina) software to account for the complex sampling design. NHANES weights were used to approximate the prevalence within the US population. Weighted means and prevalence of smell and taste impairments were determined in the total sample population, as well as within subgroups. Rao-Scott chi-squared analysis was used to determine statistical significance within subgroups. Logistic regression models were used to estimate the odds ratios and their 95% confidence intervals (95% CI) for the association of type of recent tobacco use and cigarette use with smell and taste impairments. Due to small sample sizes, nicotine product use in the last 5 days was dichotomized for modeling purposes taking into account inhaled nicotine product use. The following demographic variables were included in all adjusted analyses: age, gender, and race/ethnicity. A sensitivity analysis was conducted to identify whether those who completed the smell examination but not the taste examination differed on smoking status. This was done to examine whether the exclusion criteria introduced selection bias.

## RESULTS

### Smell impairment

Of the 3527 participants who completed the smell examination, 3186 reported on types of nicotine products (inhaled and/or smokeless) used in the last 5 days, and 13.3% failed the smell exam. There were no significant differences in type of nicotine products used in the last 5 days, cigarette use in the last 5 days, and mentholation status of regular cigarette brand according to the results of the smell exam (Table 1). Significant associations were seen between smell exam results and age (p<0.0001), and gender (p=0.0032, Table 1).

**Table 1:** Demographic and nicotine product use behavior characteristics according to the smell and taste examination results, NHANES 2013–2014

	*Smell examination*			*Taste examination*		
	*Failed N = 564*	*Passed N = 2622*	*p*	*Failed N = 495*	*Passed N = 2353*	*p*
**Age (years),** n (%)			**<0.0001**			0.1184
40–54	110 (6.8)	1115 (93.2)		220 (18.5)	919 (81.5)	
≥55	454 (18.2)	1507 (81.8)		275 (16.0)	1434 (84.0)	
**Gender,** n (%)			**0.0032**			0.3122
Male	323 (15.6)	1186 (84.4)		241 (17.6)	1137 (82.4)	
Female	241 (11.3)	1436 (88.7)		254 (16.6)	1216 (83.4)	
**Race/ethnicity,** n (%)			**0.0011**			0.0641
Non-Hispanic White	229 (12.1)	1241 (87.9)		230 (16.7)	1128 (83.3)	
Non-Hispanic Black	132 (18.0)	495 (82.0)		123 (23.0)	436 (77.0)	
Other	203 (15.8)	886 (84.2)		142 (15.4)	789 (84.6)	
**Type of nicotine products used in the last 5 days,** n (%)			0.2854			**0.0357**
No use of nicotine products	455 (13.9)	2057 (86.1)		369 (16.2)	1867 (83.8)	
Use of smokeless nicotine products only	6 (7.8)	44 (92.2)		7 (18.9)	39 (81.1)	
Use of inhaled nicotine products only	101 (11.9)	506 (88.1)		113 (19.6)	436 (80.4)	
Use of inhaled and smokeless nicotine products	2 (4.2)	15 (95.8)		6 (44.7)	11 (55.3)	
	N = 109	N = 565		N = 126	N = 486	
**Cigarette use in the last 5 days,** n (%)			0.1977			0.5834
No	19 (8.4)	90 (91.6)		18 (18.1)	84 (81.9)	
Yes	90 (11.9)	475 (88.1)		108 (21.1)	402 (78.9)	
	N = 66	N = 401		N = 95	N = 326	
**Regular brand is mentholated,** n (%)			0.4224			0.6995
No	46 (11.9)	268 (88.1)		61 (22.4)	222 (77.6)	
Yes	20 (8.4)	133 (91.6)		34 (24.5)	104 (75.5)	

Weighted frequencies are presented and were compared using the Rao-Scott chi-squared test. Bold p-values indicate statistical significance.

Those who used inhaled nicotine products with or without a smokeless nicotine product in the last 5 days had, weak and insignificant, reduced odds of failing the smell exam (OR=0.82, 95% CI: 0.52–1.29). However, after adjustment, no association was seen. Among participants who reported using an inhaled nicotine product in the last 5 days, those who used cigarettes in the last 5 days had, weak and insignificant, increased odds of failing the smell exam (OR=1.46, 95% CI: 0.76–2.82). After adjustment, this association remained insignificant (OR=1.66, 95% CI: 0.76–3.63). Among participants who reported using cigarettes in the last 5 days and who also provided a brand of cigarettes, those who provided a mentholated brand of cigarettes had slightly lower odds, although insignificant, of failing the smell examination (OR=0.68, 95% CI: 0.25–1.87). This association remained insignificant after adjustment (OR=0.57, 95% CI: 0.22–1.49, Table 2).

**Table 2:** Association of recent nicotine product use correlates and the results of the smell and taste examinations, NHANES 2013–2014

	*Smell examination*						*Taste examination*					
	*Failed*	*Passed*	*Crude*		*Adjusted^a^*		*Failed*	*Passed*	*Crude*		*Adjusted^a^*	
	*N*	*N*	*OR*	*95% CI*	*OR*	*95% CI*	*N*	*N*	*OR*	*95% CI*	*OR*	*95% CI*
**Type of nicotine products used in the last 5 days**												
Use of smokeless nicotine products only or no use of nicotine products at all	461	2101	Ref		Ref		376	1906	Ref		Ref	
Use of inhaled nicotine products with or without smokeless nicotine products	103	521	0.82	0.52–1.29	0.95	0.57–1.59	119	447	**1.35**	**1.02–1.77**	1.28	0.99–1.65
**Cigarette use in the last 5 days**												
No	19	90	Ref		Ref		18	84	Ref		Ref	
Yes	90	475	1.46	0.76–2.82	1.66	0.76–3.63	108	402	1.21	0.56–2.61	1.30	0.50–3.40
**Regular brand is mentholated**												
No	46	268	Ref		Ref		30	222	Ref		Ref	
Yes	20	133	0.68	0.25–1.87	0.57	0.22–1.49	34	104	1.12	0.59–2.14	1.12	0.55–2.30

OR: odds ratio, CI: confidence interval. Weighted logistic regressions were used to determine point estimates. Estimates in bold indicate statistical significance.

a Adjusted for age, gender and race/ethnicity.

### Taste impairment

Of the 3114 participants who completed the taste examination, 2848 reported on types of nicotine products (inhaled and/or smokeless) used in the last 5 days, and 17.1% failed the taste exam. A significant association was seen for type of nicotine products used in the last 5 days and the results of the taste exam (p=0.0357). Among participants who reported no use of nicotine products in the last 5 days, 83.8% passed the taste exam. About 81% of participants who used smokeless nicotine products only, in the last 5 days, passed the taste exam. In addition, 80.4% of participants reporting using inhaled nicotine products only, passed the taste exam. While 55.3% of participants who used inhaled and smokeless nicotine products passed the taste exam. There were no significant differences in cigarette use in the last 5 days and mentholation status of regular cigarette brand according to the results of the taste exam (Table 1).

Participants who reported using an inhaled nicotine product with or without a smokeless nicotine product in the last 5 days had significantly higher odds of failing the taste exam (OR=1.35, 95% CI: 1.02–1.77). After adjustment, this association remained, but was rendered insignificant (OR=1.28, 95% CI: 0.99–1.65). Among participants who reported using an inhaled nicotine product in the last 5 days, those who used cigarettes in the last 5 days had, weak and insignificant, increased odds of failing the taste exam (OR=1.21, 95% CI: 0.56–2.61). After adjustment, this association remained insignificant (OR=1.30, 95% CI: 0.50–3.40). No association was seen among those who reported using cigarettes in the last 5 days and also provided a mentholated brand of cigarettes, even after adjustment (Table 2). When participants who were excluded from the taste exam, but not the smell exam, were marked as all failing the taste exam or marked as all passing the taste exam (the two extreme scenarios), our findings were unaltered (data not shown).

## DISCUSSION

This study suggests that the use of nicotine products has an unclear and inconsistent relationship to dysfunctions in taste and smell. Smoking tobacco products has been linked to an increased risk of developing respiratory infections^8-10^ while inhaled and smokeless tobacco products are known to cause dental problems^10,18^, both of which are associated with chemosensory disorders^6,7^. Chemosensory disorders can greatly impact quality of life via effects on food choices, diet, nutrition, and identification of environmental hazards^5^. Our findings provide nationally representative estimates of the prevalence of smell and taste disorders by the type of nicotine product(s) recently used (e.g. inhaled and/or smokeless), recent cigarette use, and mentholation status of preferred brand of cigarettes.

The use of inhaled nicotine products, with or without smokeless nicotine products in the last 5 days, did not show any association with smell impairment^15^, but was associated with taste impairment^4,14^. On the other hand, smoking cigarettes in the last 5 days was found to have an association with smell and taste impairments, consistent with previous studies^4,14^. In both cases, this could potentially be attributed to cigarette use resulting in sinus and respiratory infections^8,9^, or alterations to taste buds and the vascularization of the fungiform papillae^12,13^. Further, the use of mentholated cigarettes approached lower odds of smell impairment and may be the result of menthol subjectively increasing nasal airflow and patency^20^, but no association was found with taste impairment. Comparison with previous studies is challenging due to differences in categorization of cigarette use (e.g. pack-years, current/former/never) and type of data used to define chemosensory impairment (e.g. examination data versus self-report). In addition, and to our knowledge, this is the first study that has looked into the effects on the chemosenses of the use of inhaled versus smokeless nicotine products.

One strength of this study is that it provided nationally representative data collected using standardized procedures and questionnaires. It does not appear that any selection bias was introduced because of the exclusion criteria. Those who completed the smell examination but did not complete the taste examination showed no differences in lifetime smoking status, nicotine product use in the last 5 days, menthol status, or cigarettes per day. However, there are some limitations to note. First, the study design is limited in that the questionnaires are at risk of recall bias, and the cross-sectional design prevents assessment of temporality, individual change in smell and taste impairments, or change in nicotine product use. Second, wide confidence intervals are partially a result of small sample sizes and indicate variability in the data. In addition, due to small sample sizes, we were unable to look at the use of specific nicotine products, individually or specific combinations with other products (e.g. cigars, smokeless) with respect to chemosensory impairments. Furthermore, the mentholation status of participant’s regular brand of cigarettes was only available for those who had smoked 100 cigarettes or more in their lifetime. Finally, the smell and taste examination data were publicly available only for one wave of NHANES, 2013–2014.

## CONCLUSIONS

Using data from NHANES 2013–2014, it was found that continued use of nicotine products could be a potential risk factor for chemosensory impairments. Although findings from this study were not statistically significant, they are suggestive of potential relationships with inhaled nicotine product use and taste impairments, and cigarette use and smell impairments, that should be examined in larger studies. Taste and smell are important chemosenses that affect quality of life and represent a first line of defense against environmental hazards. Therefore, reducing inhaled nicotine product use or quitting smoking may reduce a person’s risk of developing smell and taste impairments.

## CONFLICTS OF INTEREST

M. Goniewicz reports personal fees from Johnson & Johnson, outside the submitted work. R. J. O’Connor reports grants from National Cancer Institute, during the conduct of the study and personal fees and non-financial support from Food and Drug Administration and from World Health Organization outside the submitted work. The rest of the authors have also completed and submitted an ICMJE form for disclosure of potential conflicts of interest. The authors declare that they have no competing interests, financial or otherwise, related to the current work.
